# Biochemical and genetic approaches to the prenatal diagnosis of propionic acidemia in 78 pregnancies

**DOI:** 10.1186/s13023-020-01539-w

**Published:** 2020-10-07

**Authors:** Mengyao Dai, Bing Xiao, Huiwen Zhang, Jun Ye, Wenjuan Qiu, Hong Zhu, Lei Wang, Lili Liang, Xia Zhan, Wenjun Ji, Yu Wang, Yongguo Yu, Xuefan Gu, Lianshu Han

**Affiliations:** 1 Department of Pediatric Endocrinology and Genetic Metabolism, Xinhua Hospital, Shanghai Institute of Pediatric Research, School of Medicine, Shanghai Jiao Tong University, 1665 KongJiang Road, Shanghai, 200092 China; 2 Center for Prenatal Diagnosis, Xinhua Hospital, School of Medicine, Shanghai Jiao Tong University, Shanghai, China

**Keywords:** Propionic acidemia, Prenatal diagnosis, Amniotic fluid, Metabolite analysis, Propionylcarnitine

## Abstract

**Background:**

Propionic acidemia (PA) is a serious metabolic disorder, and different approaches have been applied to its prenatal diagnosis. To evaluate the reliability and validity of a biochemical strategy in the prenatal diagnosis of PA, we conducted a retrospective study of our 11-year experiences at a single center.

**Methods:**

We accumulated data from 78 pregnancies from 58 families referred to our center and provided prenatal diagnosis by directed genetic analysis and/or metabolite measurement using tandem mass spectrometry (MS/MS) and gas chromatography/mass spectrometry (GC/MS) of amniotic fluid (AF) samples.

**Results:**

Sixty-five unaffected fetuses (83.33%) and 13 affected fetuses (16.67%) were confirmed in our study. The characteristic metabolites including propionylcarnitine (C3) level, C3/acetylcarnitine (C2) ratio and 2-methylcitric acid (2MCA) level in unaffected and affected groups showed significant differences (*P* < 0.0001), while the level of 3-hydroxypropionic acid (3HPA) showed no significant difference between the two groups (*P* > 0.05).Of the 78 pregnancies, 24 fetuses were found to have either one causative pathogenic variant or were without genetic information in the proband. Three of these fetuses had elevated AF levels of C3, C3/C2 ratio, and 2MCA and, thus, were determined to be affected, while the remaining fetuses were determined to be unaffected based on a normal AF metabolite profile. Our genetic and biochemical results were highly consistent with postnatal follow-up results on all unaffected fetuses.

**Conclusions:**

We conclude that a biochemical approach can serve as a fast and convenient prenatal diagnostic method for pregnancies at an increased risk for PA, which could be used in conjunction with genetic testing for precise prenatal diagnosis of this disorder. In our analysis, the characteristic metabolites C3 level, C3/C2 ratio, and 2MCA level in AF supernatant were dependable biochemical markers for diagnosis, of which the C3/C2 ratio appears to be the most reliable biochemical marker for the prenatal diagnosis of PA.

## Introduction

Propionic acidemia (PA, OMIM #606054) is an organic acidemia attributed to the deficiency of propionyl-CoA carboxylase (PCC, EC6.4.1.3). PCC, which catalyzes the carboxylation of propionyl-CoA to D-methylmalonyl-CoA [1, 2], is a 750 kDa heterododecamer composed of 6 propionyl-CoA carboxylase alpha (PCCA) and 6 propionyl-CoA carboxylase beta subunits (PCCB) [3, 4]. Propionyl-CoA has a broad influence on metabolism, including impacts on the urea cycle, the citric acid cycle, and the glycine cleavage system [5]. PA has an autosomal recessive mode of inheritance and results from bi-allelic variants in *PCCA* or *PCCB* that impair enzyme function [6].

Although there are mild, late-onset forms of PA, most patients have a neonatal presentation with life-threatening metabolic decompensations [1, 7, 8]. Reliable methods for prenatal diagnosis are thus essential for neonatal management of PA. Different approaches have been applied to prenatal diagnosis of PA including direct propionyl-CoA carboxylase activity assay in chorionic villi (CV) [9, 10], quantification of the characteristic metabolites including acylcarnitines, propionic acids and methylcitric acids in cell-free amniotic fluid (AF) [11–14]; and direct pathogenic variant analysis of *PCCA* or *PCCB* genes in amniocytes. However, each approach has its limitations. Enzymatic assay can be time-consuming or unavailable, and activity can be influenced by the quality of the CV sample. Direct genetic analysis of amniocytes is dependent upon the availability of known familial mutations and diagnostic metabolites for PA in the AF can degrade over 1–2 weeks. Therefore, the use of two or more methods simultaneously may be needed to achieve the definitive prenatal diagnosis [15].

In the present study, we provide a retrospective review of our experience with prenatal diagnosis of PA over the last 11 years. Overall, we have investigated 78 pregnancies in 58 unrelated families by molecular genetics analysis of amniocytes and/or metabolite analysis in AF supernatants.

## Materials and methods

### Families and probands

In this study, 78 pregnancies (58 families) in which the probands diagnosed with PA were referred to our center seeking prenatal diagnosis from April 2008 to December 2019. The probands were diagnosed based on clinical symptoms, biochemical results and genetic testing of the *PCCA* or *PCCB* genes. Biochemical results included elevated blood levels of propionylcarnitine (C3) and propionylcarnitine to acetylcarnitine ratio (C3/C2) and elevated urine levels of 3-hydroxypropionic acid (3HPA) and 2-methylcitric acid (2MCA). The mutation spectrum of the *PCCA* and *PCCB* genes in this study is shown in Tables 1 and 2 and Table S2 [16–23]. Written informed consent was obtained from all participants, and our study was approved by the Ethics Committee of Xinhua Hospital.

**Table 1:** The prenatal results of genetic tests and biochemical analysis in the amniotic fluid samples of 13 affected fetuses

Fetus ***No.***	Variants of the proband			Variants of fetus		Metabolite of amniotic fluid			
	(PCCA: NM_000282.3; PCCB: NM_000532.3)					MS/MS		GS/MS	
	Gene	Paternal	Maternal	Fetus status	Variants origin	C3 (μmol/L)	C3/C2	3HPA (mmol/mol Cr)	2MCA (mmol/mol Cr)
F001	*PCCA*	c.1850 T > C [16]	c.1102G > C [16]	ND	ND	**6.06**	**0.62**	9.57	**2.05**
F009	*PCCA*	c.1429 + 2 T > C	Exon6del	Affected	Parental	**43.69**	**1.33**	21.20	**1.27**
F029	*PCCA*	c.1118 T > A [17]	c.1863delA	Affected	Parental	**7.44**	**0.70**	1.90	**0.87**
F069	*PCCA*	c.1118 T > A [17]	c.1118 T > A [17]	Affected	Parental	**16.55**	**0.92**	1.10	**0.74**
F034	*PCCB*	c.31_40del [18]	c.733G > A [16]	Affected	Parental	**10.66**	**1.06**	25.18	**3.56**
F035	*PCCB*	c.146delG [16]	c.838dupC [16]	Affected	Parental	**11.45**	**1.00**	8.36	**4.58**
F052	*PCCB*	c.838dupC [16]	c.167_179delinsC [16]	Affected	Parental	**29.66**	**0.83**	0.00	**1.19**
F060	*PCCB*	c.1228C > T [19]	c.838dupC [16]	Affected	Parental	**13.33**	**2.11**	3.65	**1.04**
F070	*PCCB*	c.1196C > G	Exon1-8del	Affected	Parental	**25.10**	**1.21**	2.94	**0.62**
F073	*PCCB*	c.1087 T > C [20]	c.1087 T > C [20]	Affected	Parental	**10.15**	**0.69**	0.00	**1.15**
F074	*PCCB*	c.1220del	c.167_179delinsC [16]	Affected	Parental	**5.07**	**0.63**	0.00	**0.61**
F017	ND	ND	ND	ND	ND	**9.40**	**1.43**	1.51	**2.73**
F023	ND	ND	ND	ND	ND	**15.83**	**1.39**	5.02	**2.32**
Reference range						< 5.0	< 0.3	< 35	

Elevated metabolites are shown in bold

*ND* Not determined, *C3* Propionylcarnitine, *C3/C2* C3/ acetylcarnitine (C2), *2MCA* 2-methylcitric acid, *3HPA* 3-hydroxypropionic acid, *MS/MS* Tandem mass spectrometry, *GS/MS* Chromatography/mass spectrometry

**Table 2:** Prenatal results of biochemical analysis in the amniotic fluid samples of 7 unaffected fetuses with one elevated metabolite levels

Fetus ***No.***	Variants of the proband			Variants of fetus		Metabolite of amniotic fluid			
	(PCCA: NM_000282.3; PCCB: NM_000532.3)					MS/MS		GS/MS	
	Gene	Paternal	Maternal	Fetus status	Variants origin	C3 (μmol/L)	C3/C2	3HPA (mmol/mol Cr)	MCA (mmol/mol Cr)
F008	*PCCA*	c.1429 + 2 T > C	Exon6del	ND	ND	**5.87**	0.24	3.75	0.00
F042	*PCCA*	c.2002G > A [21]	c.872C > T [22]	Normal	–	2.06	0.07	**41.28**	0.00
F043	*PCCA*	ND	c.1284 + 1G > A [23]	ND	ND	2.77	0.17	**44.42**	0.19
F053	*PCCA*	c.1330dup	c.803G > T	Carrier	Paternal	**5.00**	0.18	4.93	0.00
F044	*PCCB*	c.224A > C [16]	ND	ND	ND	1.040	0.07	**80.65**	0.00
F014	ND	ND	ND	ND	ND	1.01	0.08	**43.86**	0.00
F020	ND	ND	ND	ND	ND	0.92	0.12	2.37	**0.52**
Reference range						< 5.0	< 0.3	< 35	< 0.5

Elevated metabolites are shown in bold

*ND* Not determined, *C3* Propionylcarnitine, *C3/C2* C3/ acetylcarnitine (C2), *2MCA* 2-methylcitric acid, *3HPA* 3-hydroxypropionic acid, *MS/MS* Tandem mass spectrometry, *GS/MS* Chromatography/mass spectrometry

### Amniocyte samples

Thirty milliliters of AF were obtained from each pregnant woman by amniocentesis performed between 16 and 20 weeks of gestation. Ten mL was used for DNA extraction and the remaining supernatant from this aliquot was used for the metabolite analysis. Twenty mL of AF was cultured for karyotype analysis, with the cultured amniocytes also available as a back-up.

### Metabolite analysis of amniotic fluid

The levels of C3, C2 and C3/C2 were measured by MS/MS (Applied Biosystems, API 2000) using 3 μL of AF supernatant and the levels automatically calculated based on the assigned values for internal standards using ChemoView v1.2 software [24]. The organic acids levels in 2 mL of uncultured AF supernatant samples were measured using GC-MS (QP2010, Shimadzu Limited, Kyoto, Japan) as described by Hasegawa et al. [25].

### Gene variant analysis

DNA from AF was extracted with a QIAamp DNA Blood Mini Kit (Qiagen Inc., Valencia, CA). We then conducted polymerase chain reaction (PCR) amplification and direct Sanger sequencing. Reference sequences *PCCA* (NM_000282.3) and *PCCB* (NM_000532.3) were obtained from the NCBI GENEBANK.

### Linkage analysis

We collected 3–4 mL of peripheral blood from all PA pedigree members to perform a linkage analysis and to exclude maternal-cell contamination. Six short tandem repeats (STR) were selected according to informative STR loci in our laboratory, and we subsequently conducted the linkage analysis (STR and primers are listed in Table S1).

### Statistical analysis

Reference ranges for the four analytes evaluated in this study were determined by a nonparametric approach, identifying the 99.5th percentiles of the cumulative levels from unaffected fetuses as the upper reference limit (C3: < 5 μmol/L; C3/C2: < 0.3; Propionic acid: < 35 mmol/mol Cr; 2MCA: < 0.5 mmol/mol Cr). Standard scatter plots were generated for level of C3, C3/C2 ratio, 2MCA and 3HPA in AFs. Wilcoxon rank sum tests with exact *P* values were performed to compare the C3, C3/C2 ratio, 2MCA and 3HPA level in affected and unaffected groups. *P* < 0.05 was considered statistically significant. Statistical analysis was performed using Prism 8 (GraphPad Software Inc.).

## Results

We summarized the genetic and biochemical results of 78 prenatal samples from 58 families in our study (Table 1, Table S2), including 65 unaffected (83.33%) and 13 affected fetuses (16.67%). We found no sample with maternal contamination and uncovered no symptoms of PA upon postnatal follow-up in any of the 65 unaffected fetuses. For the 13 affected fetuses, however, the parents chose to terminate their pregnancies.

### Biochemical analyses of amniotic fluid metabolites

**Fig. 1:**
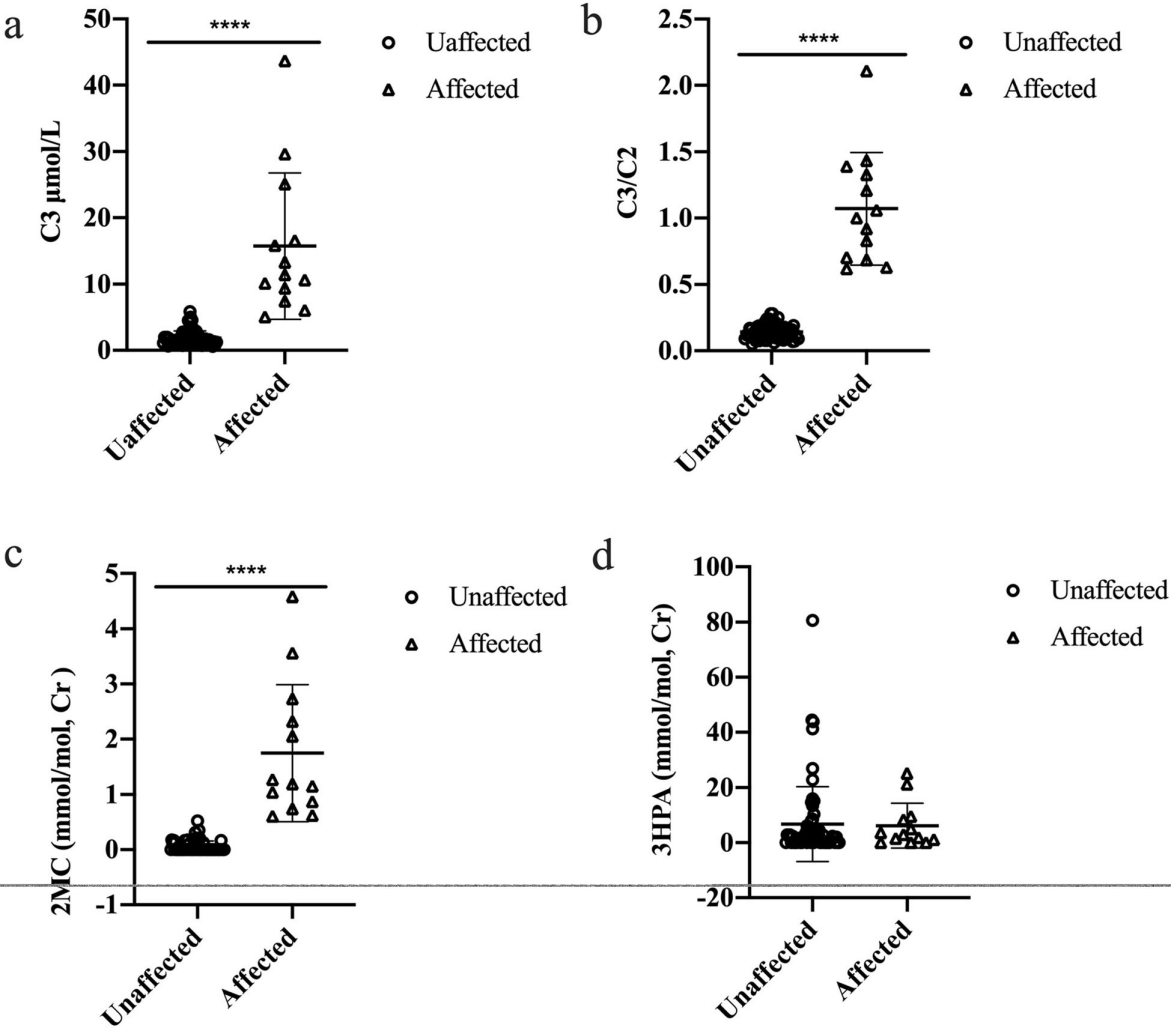
Scatter-plot showing the distribution of characteristic metabolite levels in affected and unaffected amniotic fluids. **a** The distribution of C3 levels in AFs between affected and unaffected samples; **b** the distribution of C3/C2 ratios in AFs between affected and unaffected samples; **c** the distribution of 2MCA levels in AFs between affected and unaffected samples; **d** the distribution of 3HPA in AFs between affected and unaffected samples. Horizontal lines, median values. *P* values were determined by the Wilcoxon rank sum test, *P < 0.05, ** *P* < 0.01, *** *P* < 0.001

Of 78 fetuses, 13 fetuses were determined to be affected based on metabolite tests (Table 1). The median (range) level of C3, C3/C2 ratio and 2MCA in affected samples were 11.45 μmol/L (5.07–43.69), 1.00 (0.62–2.11) and 1.19 mmol/mol Cr (0.61–4.58), respectively (Fig. 1). All of the individual levels of these three analytes in 13 fetuses was higher than that of defined reference ranges for each metabolite. However, the 3HPA levels in all affected samples, ranging from 0 to 25.18 mmol/mol Cr which were in the normal range.

Sixty-five AF supernatant samples were determined to be unaffected according to our metabolite test results (Table S2). The median (range) level of C3, C3/C2 ratio, and propionic acid, and 2MCA in 65 unaffected samples were 1.56 μmol/L (0.61–5.87), 0.14 (0.05–0.28), 2.31 mmol/mol Cr (0–80.65), and 0 mmol/mol Cr (0–0.52), respectively (Fig. 1). There were seven unaffected fetuses that had a slightly increased level of one of the four biochemical markers studied (Table 2). We noted two fetuses (F008 and F053) with slightly higher levels of C3, while the C3/C2 ratio and levels of 3HPA and 2MCA were in the normal range. Four fetuses (F014, F042, F043, and F044) were found to possess higher levels of propionic acid, but had a normal C3/C2 ratio and C3 and 2MCA levels. We found one fetus (F020) with a slightly higher level of 2MCA, but a normal C3/C2 ratio and normal levels of C3 and propionic acid.

Although the metabolite results for the C3, C3/C2 ratio, and 2MCA in unaffected and affected groups showed significant differences (*P* < 0.0001), the level of 3HPA in AF samples showed no significant difference between the two groups (*P* > 0.05, Fig. 1).

### Genetic analysis of the pathogenic variants in amniocyte DNA

There were 54 pregnancies with clear information as to pathogenic variants in probands and parents, of which 19 pregnancies were from families affected by pathogenic variants of the *PCCA* gene, while the other 35 pregnancies were from families affected by pathogenic variation in the *PCCB* gene. Twelve fetuses carried no pathogenic variants in *PCCA* or *PCCB* (22.22%), 32 fetuses carried a heterozygous variants (59.26%), and 10 fetuses carried compound heterozygous variants (18.52%; Tables 1 and 2, Table S1). To explore the relationship between genotype and levels of metabolites in AF, we compared the median level of C3, C3/C2 ratio, and propionic acid and 2MCA in *PCCA* pathogenic variant carriers (*n* = 12) or *PCCB* pathogenic variant carriers (*n* = 20) with reference samples and observed that neither *PCCA* nor *PCCB* pathogenic variant carriers showed significant differences in these four metabolite levels relative to the reference group.

Genetic and metabolite analyses of the 54 fetuses showed high consistency. Ten of 54 fetuses have been determined to be affected fetuses by molecular genetics analysis (Table 1). Metabolite analyses in AF were consistent with these results. AF samples in the 10 affected fetuses had elevated C3 level, C3/C2 ratio, and 2MCA level and we therefore also inferred them to be affected fetuses by metabolite analysis (Table 1). Forty-three of 54 fetuses were determined to be unaffected by genetic testing, including 31 carriers and 12 fetuses without identifiable mutations with normal levels of C3, C3/C2 ratio and 2MCA. There was only one fetus with inconsistent molecular genetics and metabolite results (Table 2). The fetus (F053) with a pathogenic variant inherited from the father had an AF C3 level at the cutoff of (C3 5.00 μmol/L). However, the other diagnostic metabolic markers were within normal limits. Thus, the overall metabolite panel is indicative of an unaffected fetus, as supported by molecular testing.

There were 24 fetuses with incomplete molecular genetics results. Three of these cases (F001, F017 and F023) had elevated C3, C3/C2 ratio and 2MCA in the AF and were thus determined to be affected (Table 1). The other 21 fetuses were determined to be unaffected by a normal metabolite profile, which was confirmed by a normal phenotype at postnatal follow-up. Of the 21 unaffected fetuses by biochemical testing, there were three fetuses (F014, F043 and F044) that showed high levels of propionic acid, but normal levels of the key diagnostic markers, were determined to be unaffected (Table 2 and S2). While fetus F008 was found to have a slightly elevated C3 level, the other diagnostic markers were normal, thereby determined to be unaffected. Similarly, fetus F020 had a only slightly higher 2MCA level, but with normal C3 and C3/C2 levels, was deteremined to be unaffected. Postnatal follow-ups for all 24 fetuses determined to be unaffected by biochemical testing were consistent with unaffected phenotype.

## Discussion

PA is a rare organic acidemia with an average estimated incidence of ~ 1:100,000–150,000 in the wordwide [26, 27]. The typical presentation of PA entails metabolic decompensations, cardiac complications, chronic kidney disease, or encephalopathy [28]. Prognosis of PA is generally poor and strongly influenced by the duration of coma and the level of blood ammonia [29, 30], and severe patients without proper treatment can die in the newborn period or later. Although a series of treatment strategies have been implemented over the past decade to improve the survival rate and life quality of PA patients [5, 31, 32], they remain at high risk for life-threatening complications. Prenatal diagnosis especially in families of probands with PA, is an essential strategy for neonatal planning. In the present study, we describe our experiences with prenatal diagnosis of PA in 78 pregnancies over 11 years.

The measurement of metabolite levels in AF and stable ranges across gestational ages can assist the prenatal diagnosis of PA [33–35]. The analyses of acylcarnitines by MS/MS and organic acids by GC/MS in AF allowed us to rapidly and reliably diagnose this condition, similar to our experience with prenatal diagnosis of methylmalonic acidemia [36]. Prenatal diagnosis of PA using biochemical methods required systematic evaluation. The majority of previously reported studies were based on single case or small series cases. We analyzed AF supernatants from 78 pregnancies at risk for PA using MS/MS and GS/MS techniques and showed that the measurements of three metabolite indices (C3, C3/C2 ratio and 2MCA) allowed for fast prenatal diagnosis of PA. Importantly, this metabolite panel required only a very small amount of AF supernatant, yet provided results for multiple metabolites. The propionylcarnitine (C3) level, C3/C2 ratio and 2-methylcitric acid (2MCA) level in unaffected and affected groups showed significant differences (*P* < 0.0001). 3HPA levels were also determined, but were found not to be informative, as levels in affected and unaffected cases showed no significant difference between the two groups (*P* > 0.05). The C3/C2 ratio in AFs showed no overlap between affected and unaffected fetuses, while C3, 2MCA and 3HPA levels overlapped. For 54 fetuses with confirmed pathogenic variants in the probands, the results for the C3/C2 ratio and 2MCA were consistent with the genetic results. In contrast, the levels of 3HPA in both groups showed discrepancies with the genetic results. These results suggested that the C3 level, C3/C2 ratio, and 2MCA level in AF samples were better suited than 3HPA as metabolic markers for the prenatal diagnosis of PA and that the C3/C2 ratio appeared to be the most reliable of the four tested biochemical markers. The biochemical testing approach could provide a strong support for diagnosing PA using amniotic fluid supernatant. And in the future, we could adjust cut-off value of the parameters by taking into account the molecular genetics testing to achieve a higher specificity.

Molecular genetics testing is a vital standard for the prenatal diagnosis of PA that requires the exclusion of maternal contamination to avoid false test results. However, due to the high genetic heterogeneity or novel variants whose clinical appearance is unknown, a genetic diagnosis of PA may lead to misdiagnosis [37], and the method depends upon availability of genetic information from the proband and parents. In some PA families, only one causative pathogenic variant was found in the proband, or a genetic testing was not performed, leading to the inability to make a precise prenatal diagnosis using genetic testing alone. For these families, metabolite testing could provide fast diagnosis and enable them to make an informed decision. In our study, there were 24 fetuses with incomplete genetic results in the probands: three samples were determined to be affected according to elevated levels of C3, C3/C2 ratio, and 2MCA in the AF supernatants. The remaining 21 fetuses were inferred to be unaffected fetuses based on normal metabolite levels. Additionally, postnatal follow-up showed a normal phenotype in all unaffected fetuses. Hence, biochemical testing appears to be a suitable option in diagnosis and provides further information that can be used for genetic counseling.

## Conclusion

In summary, prenatal diagnosis of PA should be offered to families with an affected proband. Although a genetic approach is routine for the prenatal diagnosis of PA, a biochemical approach would offer an additional reliable method, especially for families with incomplete genetic results. A combined genetic and biochemical approach would comprise the strongest strategy, providing increased diagnostic accuracy.

## Supplementary information

**Additional file 1: Table S1.** STR sites and primers used for excluding maternal cell contamination. **Table S2.** The prenatal results of genetic tests and biochemical analysis in the amniotic fluid samples of 65 unaffected fetuses.

## Data Availability

All data generated or analysed during this study are included in this published article and its supplementary information files.

## Abbreviations

PA: Propionic acidemia
PCC: Propionyl-CoA carboxyla*s*e
*PCCA*: 6 propionyl-CoA carboxylase alpha subunits
*PCCB*: 6 propionyl-CoA carboxylase beta subunits
MS/MS: Tandem mass spectrometry
GC/MS: Chromatography/mass spectrometry
C3: Propionylcarnitine
C2: Acetylcarnitine
C3/C2: Propionylcarnitine to acetylcarnitine ratio
2MCA: 2-methylcitric acid
AF: Amniotic fluid
3HPA: 3-hydroxypropionic acid
CV: Chorionic villi
