# Supplementary material for: Biochemical and genetic approaches to the prenatal diagnosis of propionic acidemia in 78 pregnancies
Source: Orphanet J Rare Dis. 2020 Oct 7;15:276. doi: 10.1186/s13023-020-01539-w (PMC7539428; doi:10.1186/s13023-020-01539-w)
Supplement: Supplementary file 1 — Additional file 1: Table S1. STR sites and primers used for excluding maternal cell contamination. Table S2. The prenatal results of genetic tests and biochemical analysis in the amniotic fluid samples of 65 unaffected fetuses. [file 13023_2020_1539_MOESM1_ESM.docx]

Table S1 STR sites and primers used for excluding maternal cell contamination

| **STR site** | **Forward primer** | **Reverse primer** |
| --- | --- | --- |
| D5S435 | FAM-ACACACATGCACGCTCTCTC | CAAGAGCACAGTTTGGAGTGAG |
| D5S629 | FAM-GAGGCTGAGACAGGAGAATCG | AGGCACTTAGATAAGCATTTCTGA |
| D5S610 | FAM-TCCTGTCCTCAAGTGACCCTC | GTTTGGATAGTTAAAATTGTCCTGTTT |
| D5S351 | FAM-GGAGTTTGAGACCAGTCTAGGCAACACAGCGA | TGAGACCGAAAATGCTGATGAGCATTGCCACTTTAG |
| D12S1030 | FAM-TCCCACATTGACCTATGTAGG | AGAGTGTAAATGCTACAAGGGC |
| PAH26 | FAM-GGTGGAGGACCCTCTCTTTC | CAGTCCCATCCAAATGGTTT |

Table S2 The prenatal results of genetic tests and biochemical analysis in the amniotic fluid samples of 65 unaffected fetuses

| **Fetus *No.*** | **Variants of the proband** | | | **Variants of fetus** | | **Metabolite of amniotic fluid** | | | |
| --- | --- | --- | --- | --- | --- | --- | --- | --- | --- |
|  |  |  |  |  |  | **MS/MS** | | **GS/MS** | |
|  | **Gene** | **Paternal** | **Maternal** | **Fetus status** | **Variants origin** | **C3 (μmol/L)** | **C3/C2** | **Propionic acid (mmol/mol Cr)** | **2MCA (mmol/mol Cr)** |
| F002 | *PCCA* | c.1850 T>C ^[1]^ | c.1102G>C ^[1]^ | ND | ND | 2.18 | 0.22 | 1.59 | 0.00 |
| F003 | *PCCA* | c.1850 T>C ^[1^^]^ | c.1102G>C^[1]^ | Normal | - | 2.10 | 0.21 | 0.00 | 0.31 |
| F004 | *PCCA* | c.1850 T>C ^[1]^ | c.1102G>C ^[1]^ | ND | ND | 1.06 | 0.19 | 2.22 | 0.17 |
| F005 | *PCCA* | c.863G>A | c.2002G>A ^[2]^ | Carrier | Maternal | 1.99 | 0.22 | 2.86 | 0.00 |
| F008 | *PCCA* | c.1429+2T>C | Exon6del | ND | ND | **5.87** | 0.24 | 3.75 | 0.00 |
| F010 | *PCCA* | c.1429+2T>C | Exon6del | Carrier | Maternal | 2.58 | 0.16 | 0.00 | 0.00 |
| F013 | *PCCA* | c.2002G>A ^[2]^ | ND | ND | ND | 2.41 | 0.18 | 2.02 | 0.11 |
| F015 | *PCCA* | c.1118T>A ^[3]^ | c.1831C>T ^[4]^ | Carrier | Maternal | 1.98 | 0.16 | 0.00 | 0.35 |
| F021 | *PCCA* | c.1676G>T ^[5]^ | c.862C>T ^[6]^ | Carrier | Paternal | 2.79 | 0.28 | 2.35 | 0.21 |
| F024 | *PCCA* | c.305del ^[1]^ | c.683G>T ^[1]^ | Normal | - | 2.65 | 0.15 | 2.89 | 0.19 |
| F025 | *PCCA* | c.305del ^[1]^ | c.683G>T ^[1]^ | Carrier | Maternal | 4.56 | 0.25 | 0.00 | 0.00 |
| F021 | *PCCA* | c.1118T>A ^[3]^ | c.1863delA ^[1]^ | Carrier | Maternal | 0.73 | 0.12 | 3.88 | 0.18 |
| F022 | *PCCA* | c.541A>C^[1]^ | c.2002G>A ^[2]^ | Normal | - | 1.21 | 0.16 | 0.95 | 0.11 |
| F024 | *PCCA* | c.541A>C^[1]^ | c.2002G>A ^[2]^ | Carrier | Paternal | 1.48 | 0.09 | 0.00 | 0.00 |
| F025 | *PCCA* | c.1288C>T^[7]^ | c.937C>T ^[2]^ | Carrier | Maternal | 1.98 | 0.24 | 2.79 | 0.14 |
| F042 | *PCCA* | c.2002G>A^[2]^ | c.872C>T | Normal | - | 2.06 | 0.07 | **41.28** | 0.00 |
| F043 | *PCCA* | ND | c.1284+1G>A ^[8]^ | ND | ND | 2.76 | 0.17 | **44.42** | 0.19 |
| F046 | *PCCA* | c.1040T>A^[1]^ | c.418del ^[1]^ | Carrier | Paternal | 1.42 | 0.17 | 0.00 | 0.00 |
| F053 | *PCCA* | c.1330dup | c.803G>T | Carrier | Paternal | **5.00** | 0.18 | 4.93 | 0.00 |
| F058 | *PCCA* | ND | c.130_131insAT | ND | ND | 1.41 | 0.189 | 10.37 | 0.00 |
| F063 | *PCCA* | ND | c.638-1G>C ^[1]^ | ND | ND | 1.68 | 0.18 | 26.82 | 0.00 |
| F066 | *PCCA* | c.1676G>T ^[5]^ | c.2002G>A ^[8]^ | Carrier | Maternal | 2.89 | 0.11 | 0.00 | 0.00 |
| F067 | *PCCA* | c.2002G>A ^[8]^ | Exon9-22del | Carrier | Paternal | 2.25 | 0.14 | 0.00 | 0.00 |
| F018 | *PCCB* | c.337C>T^[9]^ | c.838dup^[1]^ | Carrier | Maternal | 0.61 | 0.10 | 1.83 | 0.00 |
| F019 | *PCCB* | c.337C>T ^[9]^ | c.838dup ^[1]^ | Carrier | Maternal | 0.78 | 0.10 | 0.00 | 0.13 |
| F027 | *PCCB* | c.366_c.372+7del ^[1]^ | c.1244T>C ^[1]^ | Carrier | Paternal | 0.65 | 0.07 | 1.04 | 0.00 |
| F032 | *PCCB* | c.1495C>T ^[]10]^ | c.167_179delinsC ^[1]^ | Carrier | Paternal | 1.12 | 0.16 | 4.46 | 0.00 |
| F036 | *PCCB* | c.145_146delGGinsC ^[1]^ | c.838dup ^[1]^ | Carrier | Paternal | 1.30 | 0.12 | 0.00 | 0.00 |
| F037 | *PCCB* | c.967-2A>T ^[1]^ | c.1091-2A>G ^[1]^ | Carrier | Maternal | 0.87 | 0.10 | 3.76 | 0.18 |
| F038 | *PCCB* | ND | c.1087T>C ^[11]^ | ND | ND | 1.12 | 0.13 | 8.02 | 0.12 |
| F039 | *PCCB* | ND | c.1495C>T ^[10]^ | ND | ND | 0.89 | 0.10 | 2.31 | 0.00 |
| F040 | *PCCB* | c.1497A>G ^[1]^ | c.838dup ^[1]^ | Carrier | Paternal | 1.16 | 0.08 | 22.9 | 0.00 |
| F041 | *PCCB* | c.1497A>G ^[1]^ | c.838dup ^[1]^ | Carrier | Paternal | 1.01 | 0.08 | 2.31 | 0.00 |
| F044 | *PCCB* | c.224A>C ^[1]^ | ND | ND | ND | 1.04 | 0.08 | **80.65** | 0.00 |
| F045 | *PCCB* | c.1253C>T ^[1]^ | c.838dupC ^[1]^ | Carrier | Paternal | 0.82 | 0.08 | 0.00 | 0.00 |
| F047 | *PCCB* | c.337C>T ^[9]^ | c.866G>C ^[1]^ | Carrier | Maternal | 1.02 | 0.10 | 0.00 | 0.2 |
| F048 | *PCCB* | c.337C>T ^[9]^ | c.866G>C ^[1]^ | Carrier | Paternal | 1.31 | 0.06 | 0.00 | 0.00 |
| F049 | *PCCB* | c.839del | c.167_179delinsC ^[1]^ | Carrier | Paternal | 1.23 | 0.10 | 4.26 | 0.00 |
| F050 | *PCCB* | c.31_40del ^[12]^ | c.838dup ^[1]^ | Carrier | Maternal | 2.98 | 0.23 | 3.3 | 0.00 |
| F051 | *PCCB* | c.838dup ^[1]^ | c.167_179delinsC ^[1]^ | Carrier | Paternal | 2.26 | 0.15 | 3.67 | 0.00 |
| F054 | *PCCB* | c.838dup ^[1]^ | c.1087T>C ^[11]^ | Normal | - | 1.56 | 0.10 | 14.68 | 0.00 |
| F055 | *PCCB* | c.838dup ^[1]^ | c.1087T>C ^[11]^ | Normal | - | 1.78 | 0.14 | 1.25 | 0.00 |
| F056 | *PCCB* | c.838dup ^[1]^ | c.331C>T | Normal | - | 2.31 | 0.18 | 15.17 | 0.00 |
| F057 | *PCCB* | c.1495C>T | c.1087T>C ^[11]^ | Normal | - | 1.43 | 0.12 | 8.56 | 0.00 |
| F059 | *PCCB* | c.331C>T ^[13]^ | c.1087T>C ^[11]^ | Normal | - | 1.64 | 0.14 | 16.00 | 0.00 |
| F061 | *PCCB* | c.1228C>T ^[14]^ | c.838dup ^[1]^ | Carrier | Paternal | 1.84 | 0.10 | 13.40 | 0.00 |
| F062 | *PCCB* | c.838dup ^[1]^ | c.1087T>C ^[11]^ | Carrier | Paternal | 1.07 | 0.13 | 3.74 | 0.00 |
| F064 | *PCCB* | c.763+1G>A | c.1234G>C | Carrier | Paternal | 1.50 | 0.15 | 2.86 | 0.00 |
| F065 | *PCCB* | c.167_179delinsC ^[1]^ | c.105-2A>G | Carrier | Maternal | 2.35 | 0.12 | 1.14 | 0.00 |
| F068 | *PCCB* | c.1228C>T ^[14]^ | c.838dup ^[1]^ | Normal | - | 4.51 | 0.17 | 0.00 | 0.00 |
| F071 | *PCCB* | c.1196C>G | Exon1-8del | Normal | Maternal | 0.74 | 0.05 | 0.00 | 0.00 |
| F072 | *PCCB* | c.331C>T ^[13]^ | c.1228C>T ^[14]^ | Carrier | Paternal | 1.73 | 0.11 | 0.00 | 0.00 |
| F075 | *PCCB* | c.1535G<A | c.1535G<A | Carrier | Maternal | 4.17 | 0.09 | 0.00 | 0.00 |
| F076 | *PCCB* | c.428A>T | c.839_840insC | Normal | - | 1.20 | 0.10 | 0.00 | 0.00 |
| F077 | *PCCB* | c.391G>C ^[15]^ | c.184-2A>G | ND | ND | 1.01 | 0.12 | 0.00 | 0.00 |
| F078 | *PCCB* | c.391G>C ^[15]^ | c.184-2A>G | ND | ND | 1.16 | 0.11 | 0.00 | 0.00 |
| F006 | ND | ND | ND | ND | ND | 2.89 | 0.28 | 1.69 | 0.00 |
| F007 | ND | ND | ND | ND | ND | 1.57 | 0.23 | 5.36 | 0.17 |
| F011 | ND | ND | ND | ND | ND | 3.40 | 0.18 | 6.16 | 0.00 |
| F012 | ND | ND | ND | ND | ND | 1.33 | 0.17 | 1.22 | 0.00 |
| F014 | ND | ND | ND | ND | ND | 1.01 | 0.08 | **43.86** | 0.00 |
| F016 | ND | ND | ND | ND | ND | 1.59 | 0.16 | 2.04 | 0.15 |
| F020 | ND | ND | ND | ND | ND | 0.92 | 0.12 | 2.37 | **0.52** |
| F022 | ND | ND | ND | ND | ND | 0.99 | 0.09 | 5.12 | 0.17 |
| F026 | ND | ND | ND | ND | ND | 1.71 | 0.18 | 1.23 | 0.00 |
| Reference range | |  |  |  |  | <5.0 | <0.3 | <35 | <0.5 |

ND: not determined; C3: propionylcarnitine; C2/C3: C3/ acetylcarnitine (C2); 2MCA: 2-methylcitric acid; MS/MS: tandem mass spectrometry; GS/MS: chromatography/mass spectrometry; elevated metabolites are shown in bold.

**References**

1. Hu YH, Han LS, Ye J, Qiu WJ*, et al.* Gene mutation analysis in patients with propionic acidemia. *Zhonghua Er Ke Za Zhi* 2008;46(6):416-20.

2. Campeau E, Dupuis L, Leon-Del-Rio A, Gravel R. Coding sequence mutations in the alpha subunit of propionyl-CoA carboxylase in patients with propionic acidemia. *Mol Genet Metab* 1999;67(1):11-22.

3. Richard E, Desviat LR, Perez B, Perez-Cerda C*, et al.* Genetic heterogeneity in propionic acidemia patients with alpha-subunit defects. Identification of five novel mutations, one of them causing instability of the protein. *Biochim Biophys Acta* 1999;1453(3):351-8.

4. Sanchez-Alcudia R, Perez B, Ugarte M, Desviat LR. Feasibility of nonsense mutation readthrough as a novel therapeutical approach in propionic acidemia. *Hum Mutat* 2012;33(6):973-80.

5. Ohura T, Narisawa K, Iinuma K. Mutation analysis of the propionyl-CoA carboxylase alpha-subunit gene in four Japanese patients with propionic acidaemia. *J Inherit Metab Dis* 1999;22(7):851-2.

6. Lianou D, Gallego L, Michelakakis H, Perez-Cerda C*, et al.* Functional analysis of a novel mutation in the PCCA gene identified in a late-infantile onset propionic acidemia patient. *Clin Chim Acta* 2010;411(17-18):1388-9.

7. Nizon M, Ottolenghi C, Valayannopoulos V, Arnoux JB*, et al.* Long-term neurological outcome of a cohort of 80 patients with classical organic acidurias. *Orphanet J Rare Dis* 2013;8:148.

8. Campeau E, Desviat LR, Leclerc D, Wu X*, et al.* Structure of the PCCA gene and distribution of mutations causing propionic acidemia. *Mol Genet Metab* 2001;74(1-2):238-47.

9. Brosch S, Rauffeisen A, Baur M, Michels L*, et al.* Propionic acidemia and sensorineural hearing loss: is there a connection at the molecular genetics level. *HNO* 2008;56(1):37-42.

10. Ohura T, Narisawa K, Tada K. Propionic acidaemia: sequence analysis of mutant mRNAs from Japanese beta subunit-deficient patients. *J Inherit Metab Dis* 1993;16(5):863-7.

11. Chen Z, Wen P, Wang G, Hu Y*, et al.* Analysis of PCCA and PCCB gene mutations in patients with propionic acidemia. *Zhonghua Yi Xue Yi Chuan Xue Za Zhi* 2015;32(1):26-30.

12. Kim SN, Ryu KH, Lee EH, Kim JS*, et al.* Molecular analysis of PCCB gene in Korean patients with propionic acidemia. *Mol Genet Metab* 2002;77(3):209-16.

13. Levesque S, Lambert M, Karalis A, Melancon S*, et al.* Short-term outcome of propionic aciduria treated at presentation with N-carbamylglutamate: a retrospective review of four patients. *JIMD Rep* 2012;2:97-102.

14. Tahara T, Kraus JP, Ohura T, Rosenberg LE*, et al.* Three independent mutations in the same exon of the PCCB gene: differences between Caucasian and Japanese propionic acidaemia. *J Inherit Metab Dis* 1993;16(2):353-60.

15. Rodriguez-Pombo P, Hoenicka J, Muro S, Perez B*, et al.* Human propionyl-CoA carboxylase beta subunit gene: exon-intron definition and mutation spectrum in Spanish and Latin American propionic acidemia patients. *Am J Hum Genet* 1998;63(2):360-9.
